# Overexpression of *PgCBF3* and *PgCBF7* Transcription Factors from Pomegranate Enhances Freezing Tolerance in *Arabidopsis* under the Promoter Activity Positively Regulated by PgICE1

**DOI:** 10.3390/ijms23169439

**Published:** 2022-08-21

**Authors:** Lei Wang, Sa Wang, Ruiran Tong, Sen Wang, Jianan Yao, Jian Jiao, Ran Wan, Miaomiao Wang, Jiangli Shi, Xianbo Zheng

**Affiliations:** College of Horticulture, Henan Agricultural University, Zhengzhou 450002, China

**Keywords:** cold stress, *PgCBFs*, transcriptional regulation, pomegranate

## Abstract

Cold stress limits plant growth, development and yields, and the C-repeat binding factors (CBFs) function in the cold resistance in plants. However, how pomegranate CBF transcription factors respond to cold signal remains unclear. Considering the significantly up-regulated expression of *PgCBF3* and *PgCBF7* in cold-tolerant *Punica granatum* ‘Yudazi’ in comparison with cold-sensitive ‘Tunisia’ under 4 °C, the present study focused on the two *CBF* genes. PgCBF3 was localized in the nucleus, while PgCBF7 was localized in the cell membrane, cytoplasm, and nucleus, both owning transcriptional activation activity in yeast. Yeast one-hybrid and dual-luciferase reporter assay further confirmed that PgICE1 could specifically bind to and significantly enhance the activation activity of the promoters of *PgCBF3* and *PgCBF7*. Compared with the wild-type plants, the *PgCBF3* and *PgCBF7* transgenic *Arabidopsis thaliana* lines had the higher survival rate after cold treatment; exhibited increased the contents of soluble sugar and proline, while lower electrolyte leakage, malondialdehyde content, and reactive oxygen species production, accompanying with elevated enzyme activity of catalase, peroxidase, and superoxide dismutase; and upregulated the expression of *AtCOR15A*, *AtCOR47*, *AtRD29A*, and *AtKIN1*. Collectively, *PgCBFs* were positively regulated by the upstream PgICE1 and mediated the downstream *COR* genes expression, thereby enhancing freezing tolerance.

## 1. Introduction

Among various environmental stresses, cold stress as a common abiotic stress adversely affects plant growth and development, even survivability, and significantly restricts the geographical distribution of plants and agricultural productivity [1,2]. It is estimated that the loss of 51–82% of annual crop yield was attributed to extreme cold stress globally [3]. Cold stress causes cell membrane lipid peroxidation, decreases cell membrane fluidity, impairs photosynthesis, correspondingly leading to the accumulation of electrolyte leakage, malondialdehyde (MDA), soluble sugar, and proline [2,4,5]. Furthermore, the stability of the cell membrane system is positively correlated with the cold resistance of plants [6].

Plants have developed complex biochemical and physiological mechanisms and a flexible transcriptional network regulated by a series of transcription factors to adapt to cold stress. At present, ICE (Inducer of CBF Expression)-CBF/DREB1(C-Repeat Binding Factor/DRE Binding Factor1)-COR (cold-regulated genes) is the clearest and most pivotal regulation pathway of cold adaptation at the plant transcription level [7]. ICE1, as a transcription activator of CBFs, attaches to MYC elements in the promoter of the downstream target *CBF* genes, thus increasing CBFs transcript [8,9]. CBFs, also known as DREB1, are critical for cold acclimation in higher plants [10], and regulate *COR* gene expressions via recognizing the CRT/DRE *cis*-element in the COR promoter under cold stress [1,11]. The CBFs are APETALA2/ethylene-responsive factor (AP2/ERF) transcription factors. Growing reports have proved that CBF transcription factors play important roles in plant cold tolerance, and are extensively elucidated in model plant *Arabidopsis* [10,12,13,14]. Heterology expression of *AtCBF1* enhanced the freezing tolerance in potato [15] and tomato [16]. Importantly, CBFs are isolated in some horticulture plants, which can enhance the cold tolerance under cold stress, such as lettuce [17], plum blossom [18], tea [19], longan [20], grapevine [21,22,23], apple [24], sweet cherry [25], peach [26], almond [27], and so on. Additionally, longan *DlCBF1, DlCBF2*, and *DlCBF3* exhibited differences in the expression and function in the cold-sensitive plant species, and their overexpression in *Arabidopsis* enhanced cold tolerance accompanied with the upregulated expression of *AtRD29A*, *AtCOR15A*, *AtCOR47*, and *AtKIN1*, as well as increasing proline accumulation and reducing ROS accumulation [20,28]. Two potato *CBF1* and *StCBF1* from a frost-sensitive potato variety and *ScCBF1* from a stronger frost-resistance potato variety, also influences plant growth and development, leading to the dwarf phenotype, and *ScCBF1* exhibited a stronger tolerance than *StCBF1* [29]. Additionally, the overexpression of *Arabidopsis DREB1* and *CBF3* caused dwarfed phenotypes [28,30]. Two almond *PdCBF1* and *PdCBF2* were transiently induced by abscisic acid and drought treatments, except for the involvement in cold response [27]. The overexpression of *Jatropha curcas CBF2* improved drought stress in *Nicotiana benthamiana* [31]. Regrettably, the available information to explain CBF cold stress response signaling pathway in pomegranate was very poorly reported.

Pomegranate (*Punica granatum* L.) is regarded as a ‘miracle fruit’ due to its high nutritional value and medicinal uses [32,33]. At present, pomegranate is grown commercially in China, India, Spain, the United States, and Iran [34]. Freezing injury has become one of the most crucial limiting factors in commercial pomegranate production, as pomegranate plants do not survive long below −15 °C [35]. Currently, the research on the pomegranate cold tolerance mainly focused on the pomegranate fruit during cold storage [36,37,38,39]. However, little research has been reported on the molecular mechanism of cold resistance in pomegranate. Considering that the CBF signaling pathway is still poorly understood in pomegranate, in the present study, two CBF transcription factors were identified and characterized from a cold-tolerant *P. granatum* cultivar ‘Yudazi’, which aimed to elucidate the potential mechanism of the CBF signaling pathway to improve the cold tolerance in pomegranate.

## 2. Results

### 2.1. Screening and Analyzing the Candidate CBF Genes

**Figure 1:**
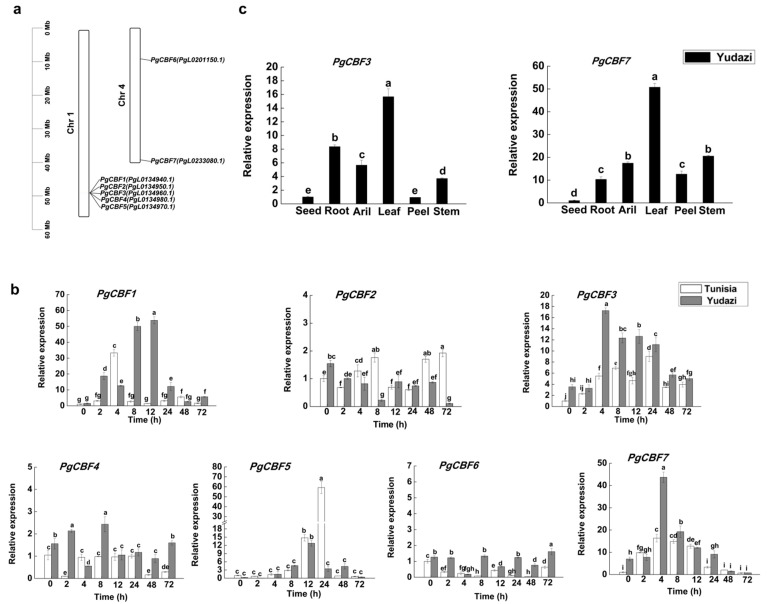
Chromosome distribution and the expression profiles of *PgCBF* gene family members from pomegranate vs. ‘Tunisia’ and ‘Yudazi’ at 4 °C. (**a**) Chromosome distribution of *CBF* genes in *Punica granatum* genome. Chr 1 and 4 represent 2 chromosomes. Gene position and chromosome length are measured with the left ruler (bp); (**b**) Expression profiles of *PgCBF* gene family members under 4 °C; (**c**) Expression patterns of *PgCBF3* and *PgCBF7* genes in six tissues from ‘Yudazi’. Different letters present significant differences (*p* < 0.05).

Potential CBF proteins were identified in pomegranate genome databases by their homology with *Arabidopsis*. Seven *PgCBF* genes were distributed on two chromosomes (Chromosome 1 and Chromosome 4) in pomegranate and named according to their positions on the chromosomes in the pomegranate genome (Figure 1a). Here, the comparison of expression patterns of *CBFs* from two pomegranate cultivars was investigated under 4 ℃ by qRT-PCR method. The results indicate that the expressions of *PgCBF3*, *PgCBF4*, *PgCBF6,* and *PgCBF7* were higher in ‘Yudazi’ than in ‘Tunisia’, especially for *PgCBF3* and *PgCBF7,* showing significant differences. Moreover, *PgCBF3* and *PgCBF7* transcript abundance both peaked at 4 h after cold treatment (Figure 1b). Accordingly, the genes *PgCBF3* and *PgCBF7* located at Chromosome 1 and Chromosome 4 were selected as the candidate genes in this study (Figure 1a).

As shown in Figure 1c, *PgCBF3* and *PgCBF7* had tissue-specificity expression in six tissues from ‘Yudazi’ with tissue specificity. The expression of *PgCBF3* revealed to be the highest in leaves, followed by the roots, and the lowest in seeds and peel. The expression of *PgCBF7* also revealed to be the highest in leaves, followed by the aril and stem, and the lowest in seeds (Figure 1c).

**Figure 2:**
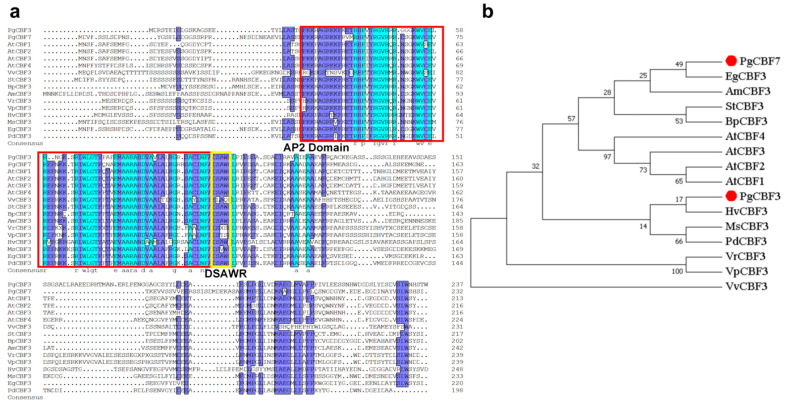
Alignment and phylogenetic analysis of PgCBF3 and PgCBF7 protein with other CBF proteins. (**a**) Sequence alignment of PgCBF3 and PgCBF7 protein with the other CBF proteins from other plant species; (**b**) Phylogenetic tree of CBF proteins from different species. AtCBF1 (AT4G25490.1); AtCBF2 (AT4G25470.1); AtCBF3 (AT4G25480.1); AtCBF4 (AT5G51990.1); BpCBF3 (QIJ58752.1); AmCBF3 (AHH86061.1); StCBF3 (ACB45095.1); PgCBF3 (PgL0134960.1); PgCBF7 (PgL0233080.1); MsCBF3 (ARO50175.1); HvCBF3 (ABE02655.1); VrCBF3 (AIL00734.1); VpCBF3 (AIL00707.1); VvCBF3 (QBC35953.1); EgCBF3 (AQX36212.1); PdCBF3 (AIQ82401.1).

The ORF sequences of *PgCBF3* and *PgCBF7* were obtained, 714 bp and 699 bp, encoding 237 and 232 amino acids, respectively (Figure S1). An alignment of the amino acid sequences of PgCBF3 and PgCBF7 with other species CBFs showed that PgCBF3 and PgCBF7 proteins contained the AP2 domain presented and DSAWR CBF signature motif (Figure 2a). PgCBF3 had a high homology with *Betula platyphylla* CBF3 (47.95%) and AtCBF3 (46.15%), while PgCBF7 had a high sequence similarity with *Eucalyptus globulus* CBF3 (58.65%) and AtCBF3 (53.85%) (Figure 2a). The sequence homology between PgCBF3 and PgCBF7 was 40.86% (Figure 2a). The phylogenetic relationship demonstrated that PgCBF3 had a close relationship *Hordeum vulgare* CBF3, while PgCBF7 did with EgCBF3 (Figure 2b).

### 2.2. Subcellular Localization and Transcriptional Activity Analysis of PgCBF3 and PgCBF7

**Figure 3:**
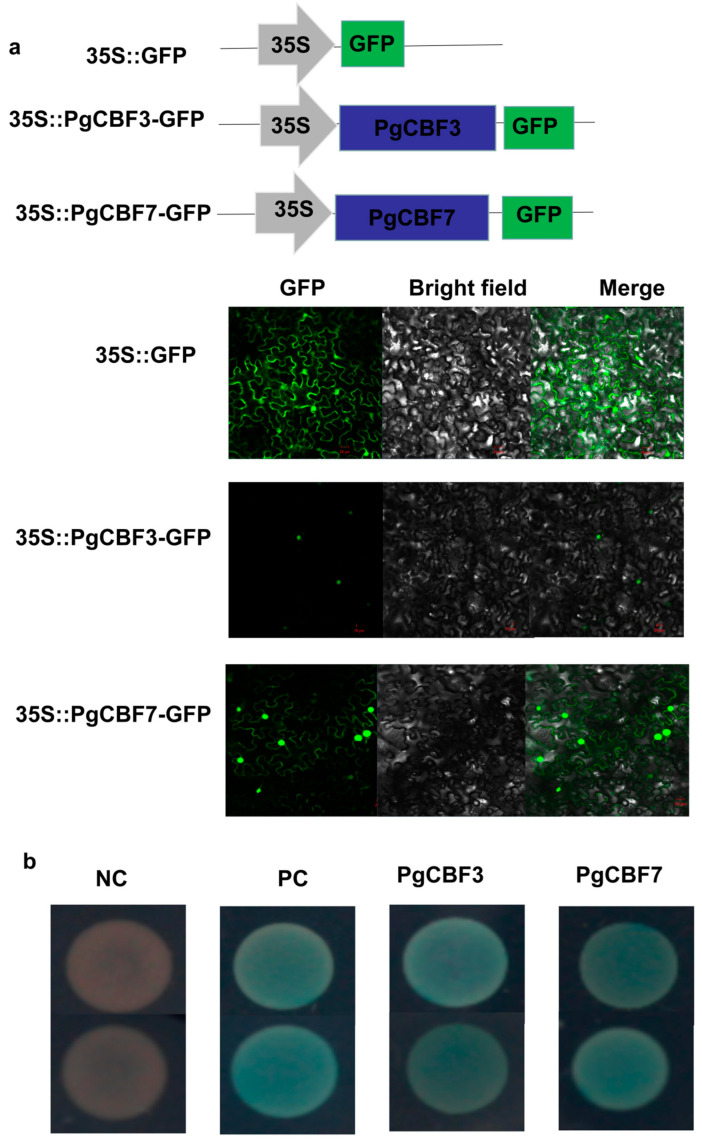
Subcellular localization and transactivation assay of PgCBF3 and PgCBF7. (**a**) Subcellular localization of PgCBF3 and PgCBF7 fusion protein in tobacco epidermal cells; GFP: Green fluorescent protein fluorescence; BF: Bright field; Merge: The fusion of green fluorescence and bright field; Bar = 20 μm; (**b**) Transcriptional activity analysis of PgCBF3 and PgCBF7 transcription factors. NC: Negative control, pGADT7-T + pGBKT7-Lam; PC: Positive control, pGADT7-T + pGBKT7-p53; PgCBF3: pGADT7-T + pGBKT7-PgCBF3; PgCBF7: pGADT7-T + pGBKT7-PgCBF7.

In order to explore the function of PgCBF3 and PgCBF7, the recombinant vectors pCAMBIA2300-35S-PgCBF3-GFP and pCAMBIA2300-35S-PgCBF7-GFP were transiently transformed into tobacco leaves. The results demonstrate that 35S::PgCBF3-GFP was located in the nucleus, 35S::PgCBF7-GFP was located in the nucleus, cell membrane, and cytoplasm, while 35S:: GFP was located in the whole cell (Figure 3a). As shown in Figure 3b, the yeast stains harboring pGBKT7-PgCBF3 and pGBKT7-PgCBF7 both grew normally on SD/-Trp-Leu-His-Ade medium with X-α-gal and turned to blue, indicating that PgCBF3 and PgCBF7 functioned as a transcription activator in yeast.

### 2.3. Validation of Activation Activity of PgICE1 on the Promoters of PgCBF3/PgCBF7

In view of the fact that a bHLH-type transcription factor AtICE1 positively regulated the expression of *CBFs* in the cold, *PgICE1* gene was cloned from *P. granatum* ‘Yudazi’, and its protein sequence showed a higher homology with ICE1 proteins from other species, sharing 49.81% of amino acid identity with AtICE1 (Figure S2a), and clustered into the same clade with EgICE1 and SoICE1 (*Syzygium oleosum*) (Figure S2b).

**Figure 4:**
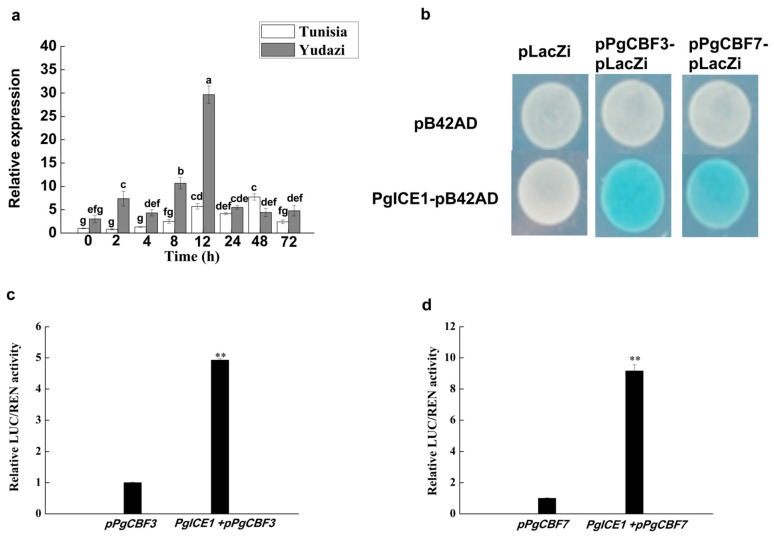
The expression profiles of *PgICE1* gene at 4 °C and yeast one-hybrid and Dual-LUC experiment of *PgICE1* on *PgCBF3* and *PgCBF7* promoter. (**a**) The expression profiles of *PgICE1* gene at 4 °C; (**b**) Yeast one-hybrid experiment of *PgICE1* on *PgCBF3* and *PgCBF7* promoter; (**c**) Dual-LUC activity of *PgICE1* and *PgCBF3* promoter; (**d**) Dual-LUC activity of *PgICE1* and *PgCBF7* promoter. Different letters present significant differences (*p* < 0.05), and ** presents significant differences (*p* < 0.01).

In order to explore the relationship of *PgICE1* on *PgCBF3* and *PgCBF7* promoters, the promoter sequences of *PgCBF3* (1991 bp) and *PgCBF7* (2000 bp) from *P. granatum* ‘Yudazi’ were analyzed (Figure S3). Using PlantCARE, some *cis*-acting elements were found in the promoters of *PgCBF3* and *PgCBF7*, both including LTR (*cis*-acting element involved in low-temperature responsiveness), MYC recognition sequences which ICE1 specifically binds to, along with ABRE and MBS (Figure S3). In addition, the *PgICE1* expression was also induced by low temperature, significantly higher in cold-tolerant cultivar ‘Yudazi’ than in cold-sensitive cultivar ‘Tunisia’, especially at 12 h after cold treatment (Figure 4a), which demonstrated that *PgICE1* as well as *PgCBF3* and *PgCBF7* all expressed at a higher level in ‘Yudazi’ under low temperature.

The binding of PgICE1 to the promoters of *PgCBF3* and *PgCBF7* were verified by yeast one-hybrid technology, indicating that PgICE1 induced the activity of the promoters of *PgCBF3* and *PgCBF7*, respectively (Figure 4b). Furthermore, PgICE1 had a stronger activation effect on the promoter sequences of the promoters of *PgCBF3* and *PgCBF7,* based on the dual-LUC reporter assay (Figure 4c,d).

### 2.4. Overexpression of PgCBF3/PgCBF7 Enhanced Freezing Tolerance in A. thaliana

**Figure 5:**
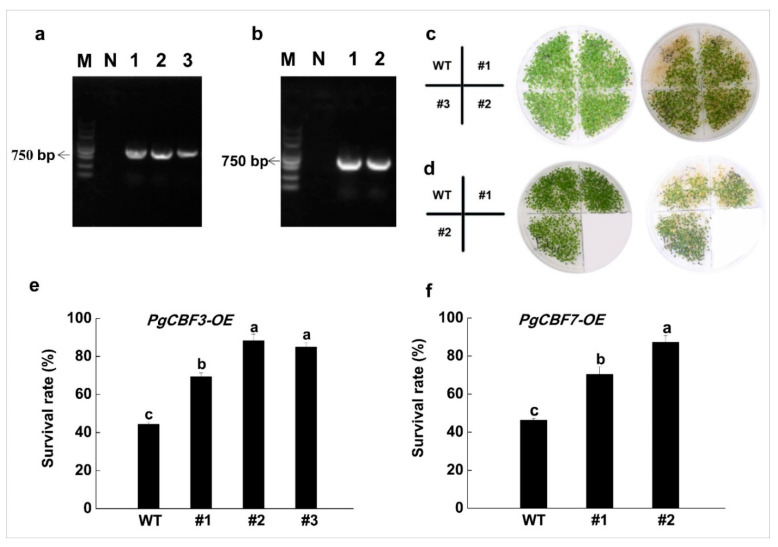
Identification of *PgCBF3* and *PgCBF7* transgenic *Arabidopsis* lines and phenotype and survival rate of *PgCBF3* and *PgCBF7* transgenic *Arabidopsis* lines at −2 °C. Identification of transgenic *Arabidopsis* lines of *PgCBF3* (**a**) and *PgCBF7* (**b**), N represents a negative control using sterilized water; phenotype of transgenic *Arabidopsis* lines of *PgCBF3* (**c**) and *PgCBF7* (**d**) after −2 °C treatment; survival rate of transgenic *Arabidopsis* lines of *PgCBF3* (**e**) and *PgCBF7* (**f**) after −2 °C. Different letters present significant differences (*p* < 0.05).

**Figure 6:**
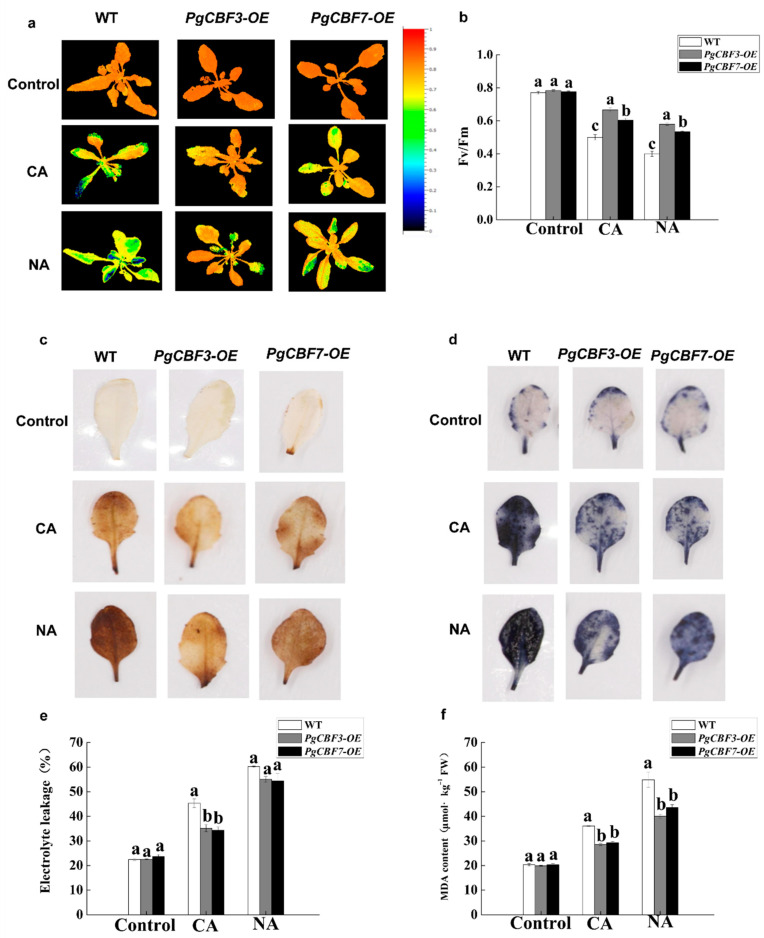
Change trends of physiological parameters of transgenic lines and WT plants under freezing treatment. The chlorophyll fluorescence image (**a**) and Fv/Fm values (**b**) of *PgCBF3* and *PgCBF7* transgenic *Arabidopsis* under freezing stress; (**c**) H_2_O_2_ accumulation by DAB chemical staining; (**d**) detection of O^2-^ by NBT chemical staining electrolyte leakage (**e**) and MDA (**f**) content of *PgCBF3* and *PgCBF7* transgenic *Arabidopsis* at freezing temperature. Different letters present significant differences (*p* < 0.05). CA: cold-acclimated; NA: non-acclimated.

To further explore the potential function of *PgCBFs* in plants, the overexpression vectors were transformed into *A. thaliana*. After PCR validation, three transgenic PgCBF3-OE lines and two transgenic PgCBF7-OE lines were obtained (Figure 5a,b). We found that the survival rate of PgCBF3-OE plants and PgCBF7-OE plants reached 80.9% and 79.2%, respectively, which is higher than 45.5% in the wild-type plants at 1 h after −2 °C treatment (Figure 5c–f). Moreover, we examined whether the freezing tolerance of the transgenic *PgCBF3* and *PgCBF7* plants was regulated by *CBF* genes under both NA and CA conditions. The results demonstrated that chlorophyll fluorescence was weaker in the leaves from the CA and NA groups than in the controls (Figure 6a). Additionally, the Fv/Fm values reduced after CA and NA treatment, but was higher in the transgenic *PgCBF3* and *PgCBF7* plants than in the wild-type plants (Figure 6b). Collectively, *PgCBF3* and *PgCBF7* genes may improve the freezing tolerance in the transgenic *A. thaliana* plants.

Subsequently, the physiological and biochemical indices were investigated between wild-type and transgenic plants under freezing stress, including the accumulation of ROS, O^2-^, and H_2_O_2_. As shown in Figure 6c,d, the DAB and NBT staining revealed that a lighter color appeared in transgenic and wild-type *Arabidopsis* plants before freezing treatment, without obvious difference. However, after CA and NA treatments, more brown or blue plaques distributed on the leaves from the wild-type ones, suggesting that less ROS accumulation presented in the transgenic lines than the wild-type plants under freezing stress. Furthermore, electrolyte leakage and MDA content were also lower in the transgenic plants than in wild-type plants after CA and NA treatments (Figure 6e,f). These results suggested that the heterologous expression of *PgCBF3* and *PgCBF7* in *A. thaliana* protected the integrity of cell membrane and reduced oxidation damage, which is beneficial for freezing tolerance.

**Figure 7:**
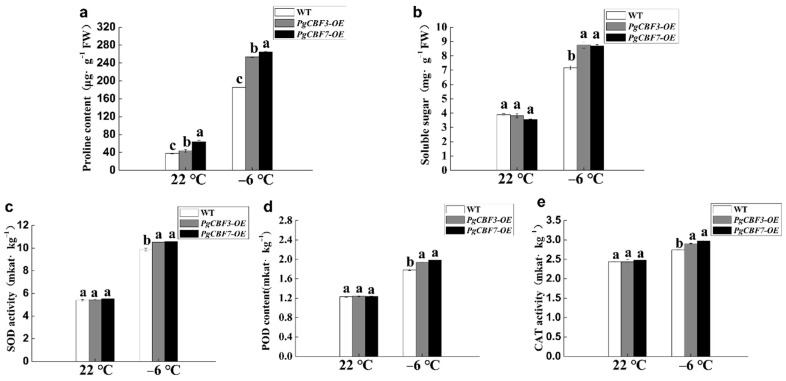
Proline (**a**), soluble sugars (**b**), and enzyme activity of SOD (**c**), POD (**d**), and CAT (**e**) in the *PgCBF3* and *PgCBF7* transgenic *Arabidopsis* at freezing temperature. Different letters present significant differences (*p* < 0.05).

**Figure 8:**
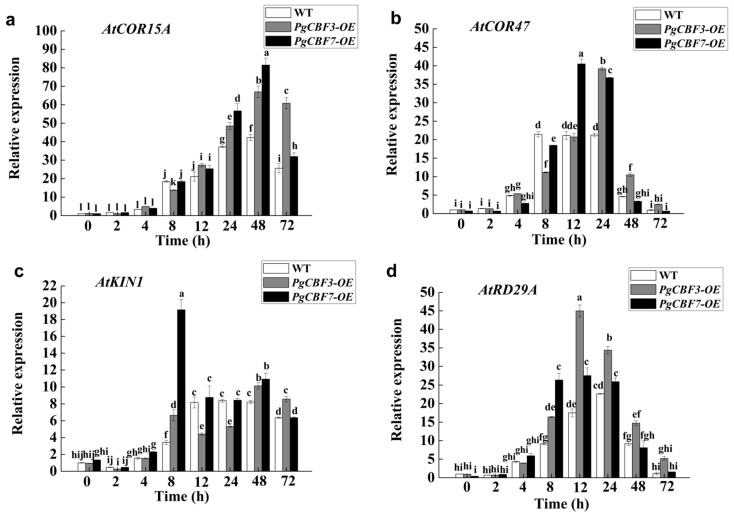
Relative expressions levels of the four cold-responsive genes *AtCOR15A* (**a**), *AtCOR47* (**b**), *AtKIN1* (**c**), and *AtRD29A* (**d**) in *PgCBF3* and *PgCBF7* transgenic *Arabidopsis* under cold stress. Different letters present significant differences (*p* < 0.05).

Generally, proline and soluble sugars are considered important compatible solutes in response to cold treatment. In the current study, the contents of proline and soluble sugars were significantly higher in PgCBF3-OE and PgCBF7-OE lines than in wild-type plants after −6 °C treatment (Figure 7a,b). The enzyme activities of SOD, POD, and CAT are also used to evaluate the freezing tolerance. It was found that they significantly accumulated in transgenic plants (PgCBF3-OE and PgCBF7-OE lines) after −6 °C treatment (Figure 7c–e). Furthermore, qRT-PCR analysis supported the finding, as the expression of *AtCOR15A*, *AtCOR47*, *AtRD29A*, and *AtKIN1* were all higher in the PgCBF3-OE and PgCBF7-OE lines than in the wild-type plants after cold treatment (Figure 8). In conclusion, our findings demonstrated that the overexpression of *PgCBF3* and *PgCBF7* in *A. thaliana* enhances cold tolerance, which was likely via the increasing expression of four cold-responsive genes.

## 3. Discussion

For perennial fruit trees, the cold tolerance is a determinant factor for survival, especially in fall and late winter, when even young plants are more sensitive to low temperatures. The prominent role of CBF transcription factor genes in response to cold stress also attracted many researchers’ attention. Plum blossom *CBFs* were higher in high cold resistance variety than in low cold resistance variety [18]. Meanwhile, three longan *DlCBF* genes exhibited differences in their expression and function [20]. Therefore, a cold-sensitive ‘Tunisia’ and a cold-tolerance ‘Yudazi’ were selected to screen potential *CBF* genes. The expression profiles of seven pomegranate *CBF* genes in the two pomegranate cultivars produced a satisfied result that *PgCBF3* and *PgCBF7* were significantly up-regulated expression in ‘Yudazi’ at 4 °C (Figure 1), then provided a significant theoretical basis for further study of the two *CBF* genes. Additionally, the tissue-specific expression of *PgCBF3* and *PgCBF7* revealed to be the highest in leaves, in agreement with the higher expression of *DlCBF1* and *DlCBF2* in longan young leaves [20]. Sequence analysis showed that PgCBF3 and PgCBF7 shared a high homology with *B. platyphylla* CBF3 and *E. globulus* CBF3, respectively. Furthermore, subcellular localization analysis revealed that PgCBF3 was located on the nucleus while PgCBF7 was located on the nucleus, cytoplasm, and cell membrane, and both PgCBF3 and PgCBF7 had a transcriptional activation function (Figure 3), similar to the previous results of tea CBF1-6 proteins [19] and longan DlCBF1, DlCBF2, and DlCBF3 [20].

Transcription factors are largely responsible for the selectivity in gene regulation; therefore, gene expression is mainly regulated at the transcriptional level, dependent on diverse *cis*-acting elements in the promoter [40,41]. *CBF* gene expression is modulated at the transcriptional level by various transcription factors via recognizing different *cis*-elements, including ICE1 [9,42], ICE2 [42,43], brassinazole-resistant 1 (BZR1) [44], pseudo response regulators (PRRs) [45], ethylene-insensitive 3 (EIN3) [46], etc. To explore the possible mechanisms for the two pomegranate *CBFs*, their upstream sequences were cloned. Our findings show that the promoters of *PgCBF3* and *PgCBF7* contained the LTR element and MYC recognition site which was necessary for ICE binding under cold stress (Supplementary Figure S3). Furthermore, yeast one-hybrid and Dual-LUC assay proved that PgICE1 positively regulated the expression of *PgCBF3* and *PgCBF7* genes by combining with the promoters (Figure 4), which was consistent with the result that ICE binds to the MYC *cis*-acting element in the *CBF* promoter and activates the expression of *CBFs* [3,9,47]. Collectively, PgICE1 could bind to the promoters of *PgCBF3* and *PgCBF7*, likely via the MYC recognition sequences.

Seen as transcription factors possess unique characteristics and modes of action, the overexpression strategy has been particularly effective in revealing transcription factor function [40]. Some evidence has also indicated that the heterologous expression of *CBF* genes from peach [26], longan [20], and sweet cherry [25] increased cold tolerance in apple and *Arabidopsis* plants. For −2 °C treatment, the ectopic expression of *PgCBF3* and *PgCBF7* in *Arabidopsis* improved the freezing tolerance significantly by higher survival rate and less phenotype damage, as seen from similar results in previous studies [19,48]. Moreover, the higher expression of *AtCOR15A*, *AtCOR47*, *AtKIN1,* and *AtRD29A* were presented in the PgCBF3-OE and PgCBF7-OE transgenic lines under 4 °C treatment, suggesting that *PgCBF3/7* up-regulated the expression of these target genes and increased the tolerance against low temperature damage. Although the protein sequences of PgCBF3 and PgCBF7 had no high similarity with *Arabidopsis* CBF3 (46.15% and 53.85%), and were not clustered into the same group with AtCBF1-4, PgCBF3, and PgCBF7, they played an important role in *COR* genes expression, which was attributed to *COR* genes expression regulated by ICE-CBF/DREB1 cascade under cold stress [20,28,49,50].

In addition, low temperature causes multiple biochemical and physiological changes. MDA accumulation and electrolyte leakage are widely used to evaluate membrane integrity [51,52]. In the PgCBF3-OE and PgCBF7-OE transgenic lines, the contents of electrolyte leakage and MDA both rose after CA and NA treatments, but lowered in the wild-type *Arabidopsis* plants, especially exhibiting a significant difference of MDA content with the wild-type plants. ROS production is investigated to assess the stress tolerance in response to cold stress in plants [20,52,53]. In the current study, after CA and NA treatments, ROS accumulation increased in both the transgenic lines and wild-type plants, but was strikingly lower than the wild-type plants, which was consistent with the rapidly accumulated ROS for cold acclimation [54]. Meanwhile, the ectopic expression of two Asian pear *PpyCBFs* and three longan *DlCBFs* also resulted in lower ROS accumulation [20,53]. Consequently, we detected the activity of POD, SOD, and CAT in pomegranate, responsible for the elimination of ROS in plant cells [54,55]. The findings demonstrated that higher activity of the three major ROS scavenging enzymes (CAT, SOD, and POD) existed in the transgenic lines PgCBF3-OE and PgCBF7-OE, compared with wild-type plants after −6 °C treatment, which partially accounted for less ROS accumulation in the transgenic lines. In conclusion, it was noted that the transgenic *PgCBF3* and *PgCBF7* lines acquired increased cold tolerance due to minor oxidation damage in cells and relatively stabilizing cell membranes via activating the antioxidant enzymes activity in a more robust manner. Besides, the higher concentration of soluble sugar and proline in transgenic lines greatly contributes to rising the tolerance against cold stress, which was stated in the overexpression of *Arabidopsis CBF3* [30].

## 4. Materials and Methods

### 4.1. Plant Materials and Growth Conditions

*P. granatum* vs. ‘Yudazi’ and ‘Tunisia’ plants were grown in the fruit tree experimental station of the College of Horticulture, Henan Agricultural University, Zhengzhou, China. ‘Yudazi’ has a strong tolerance to low temperature, while ‘Tunisia’ has a weak tolerance to low temperature [39]. Cuttings from ‘Yudazi’ and ‘Tunisia’ were rooted and grown in a growth chamber at 22 °C and 75% of relative moisture with a 16/8 light/dark photoperiod. Additionally, *Arabidopsis thaliana* seedlings (ecotype Columbia, Col-0) and *N. benthamiana* seedlings were grown under the same conditions.

### 4.2. Cold-Tolerance Assays

The healthy 30-day-old pomegranate cuttings and the 3-week-old *A. thaliana* plants were transferred into a growth chamber at 4 °C for cold treatment. The leaves were collected at 0, 2, 4, 8, 12, 24, 48, and 72 h after cold treatment and immediately frozen in liquid nitrogen.

Freezing tolerance assays were conducted as described by Hu et al. [56], with some modifications. The 10-day-old *Arabidopsis* seedlings were maintained for 1 h in a growth chamber at −2 °C, and transferred to 22 °C. The survival rates were measured visually after 24 h. Three-week-old *Arabidopsis* plants were divided into two groups, one group (cold-acclimated, CA) was set at 4 °C for 7 days, and −7 °C for 8 h, while the other group (non-acclimated, NA) was placed at −6 °C for 8 h.

### 4.3. RNA Extraction and Quantitative RT-PCR (qRT-PCR) Analysis

The total RNA was extracted from pomegranate and *Arabidopsis* using the Plant Total RNA Isolation Kit (Shanghai Sangon Biotech Co., Ltd., Shanghai, China), and the cDNA was synthesized using HiScript III RT SuperMix for qPCR (+gDNA wiper) (Nanjing Vazyme Biotech Co., Ltd., Nanjing, China). qRT-PCR was carried out using ChamQ Universal SYBR qPCR Master Mix (Nanjing Vazyme Biotech Co., Ltd., Nanjing, China) on ABI 7500 PCR instrument (Applied Biosystems, Foster, CA, USA). The *PgActin* (LOC116200207) and *AtActin* (AT3G18780) genes were used as an internal reference. All the primers used were listed in Supplementary Table S1.

### 4.4. Bioinformatics Analysis of PgCBF3 and PgCBF7

The protein sequences of AtCBF1 (AT4G25490), AtCBF2 (AT4G25470), AtCBF3 (AT4G25480), AtCBF4 (AT5G51990), AtCBF5 (AT1G63030), and AtCBF6 (AT1G12610) were obtained from TAIR webserver (https://www.arabidopsis.org/, accessed on 23 February 2004). Pomegranate genome annotation information, genome sequences, and protein sequences were obtained from the pomegranate genome database (Table S2) [57]. The coding sequences of 7 pomegranate *CBF* candidate genes were obtained using TBtools v.1.09876 and NCBI website (https://www.ncbi.nlm.nih.gov/) with BLAST search. Sequences of the other plant CBF proteins were retrieved using BLAST search in NCBI database (https://blast.ncbi.nlm.nih.gov/Blast.cgi, accessed on 17 March 2022). The amino acid sequence alignment was performed using DNAMAN software. A phylogenetic tree was constructed with the neighbor-joining method by MEGA6.0.

### 4.5. Histochemical Staining

The 3,3′-diaminobenzidine (DAB) and nitroblue tetrazolium (NBT) staining were used to detect hydrogen peroxide (H_2_O_2_) and superoxide anion (O^2−^), respectively, as previously described [58,59].

### 4.6. Chlorophyll Fluorescence Detection

The *Arabidopsis* plants at low temperature were imaged by a SPAD-502 chlorophyll (Chl) meter (Minolta, Tokyo, Japan), and SPAD values were recorded. The maximum quantum efficiency of PSII efficiency (Fv/Fm) was assessed using FluorCam7 Chl fluorescence imaging (Photon Systems Instruments, Brno, Czech Republic) and LI-6400 portable photosynthesis measurement system (LI-COR Inc., Lincoln, NE, USA) [60].

### 4.7. Recombination Vectors Construction and Transgenic Plants Generation

To generate the overexpression transgenic plants, the open reading frame (ORF) sequences of *PgCBF3* and *PgCBF7* (containing *KpnI* and *BamHI* digestion sites) driven by the *CaMV 35S* promoter were cloned into the pCAMBIA2300-GFP vector, generating the vectors pCAMBIA2300-35S-PgCBF3/PgCBF7-GFP. The recombinant vectors were introduced into *A. thaliana* (ecotype Columbia, Col-0) via *Agrobacterium tumefaciens* strain GV3101 using inflorescence infection method [61].

### 4.8. Determination of Electrolyte Leakage, MDA and Proline Content, and Enzyme Activity

The electrolyte leakage was evaluated according to the method described by Nanjo et al. [62]. MDA content (μmol kg^−1^ fresh weight) was measured by thiobarbituric acid method using MDA-2-Y Kit (Suzhou Comin Biotechnology Co. Ltd., Suzhou China). The enzyme activity of catalase (CAT) and superoxide dismutase (SOD) were determined using CAT-2-W Kit and SOD-2-Y Kit and (Suzhou Comin Biotechnology Co. Ltd., China), respectively. Soluble sugar content was measured using KT-1-Y Kit (Suzhou Comin Biotechnology Co. Ltd., China). The proline content was assessed using Proline (PRO) Content Assay Kit BC02905 (Beijing Solarbio Science & Technology Co., Ltd., Beijing, China). Peroxidase (POD) activity was determined according to the method described previously [63].

### 4.9. Determination of Electrolyte Leakage, MDA and Proline Content, and Enzyme Activity

The *A. tumefaciens* strain GV3101 with recombinant vectors pCAMBIA2300-35S-PgCBF3-GFP and pCAMBIA2300-35S-PgCBF7-GFP were infiltrated into tobacco leaves, the empty vector as the control. After 72 h, GFP fluorescence was observed by a confocal laser microscopy (Carl Zeiss, Jena, Germany).

### 4.10. Transcriptional Activation Activity Analysis

The ORF sequences of *PgCBF3* and *PgCBF7* were cloned into pGBKT7. According to the procedure of Yeastmaker^TM^ Yeast transformation system 2 (Clontech, Takara, Kusatsu, Japan), the recombinants of pGBKT7-PgCBF3 and pGBKT7-PgCBF7 were transformed into the yeast strains Y2HGold stain, which were screened on SD/-Trp-Leu-His-Ade/X-α-Gal medium. The yeast with pGADT7-T + pGBKT7-Lam was used as the negative control and with pGADT7-T + pGBKT7-p53 as the positive control.

### 4.11. Extraction of Genomic DNA and the Cloning of the Promoters

DNA extraction from pomegranate leaves was performed using the EZ-10 Spin Column Plant Genomic DNA Purification Kit (Shanghai Sangon Biotech Co., Ltd., China). The purified DNA was used for the promoter amplification of *PgCBF3/PgCBF7* genes. The promoter sequences were analyzed using PLACE (https://www.dna.affrc.go.jp/PLACE/?action=newplace, accessed on 8 January 2007) and PlantCARE (http://bioinformatics.psb.ugent.be/webtools/plantcare/html/).

### 4.12. Yeast One-Hybrid Assay

The promoter fragments of *PgCBF3* and *PgCBF7* genes were inserted into the pLacZi vector, while the ORF of *PgICE1* was cloned into the pB42AD vector. After co-transformation pPgCBF3/7-pLacZi and PgICE1-pB42AD in EGY48 yeast strains, yeast cells grown on SD-Trp/Ura media were suspended in sterile water and then placed on SC-Trp/Ura/X-Gal media for culturing at 28 °C for 12–24 h.

### 4.13. Dual-Luciferase (Dual-LUC) Reporter Assay

The ORF sequence of *PgICE1* was cloned into the pSAK277 vector, which acted as the effector, and the promoters of *PgCBF3* and *PgCBF7* were cloned into the pGREENII0800-LUC, which acted as the reporter. All constructs were individually transformed into *Agrobacterium* GV3101 stain and expressed transiently in tobacco leaves by *A. tumefaciens*-mediated infiltration. After culturing for 3 days, the ratio of enzyme activities of firefly luciferase (LUC) and renilla luciferase (REN) was measured using Dual Luciferase Reporter Gene Assay Kit (Vazyme Biotech Co., Ltd., Nanjing, China).

### 4.14. Statistical Analysis

The mean values and standard deviation (SD) were obtained for at least three repetitions. Statistical analysis was performed using SPSS Statistics v.20. Significant difference was evaluated using one-sided paired *t* test (*p* < 0.05).

## 5. Conclusions

Two CBFs (*PgCBF3* and *PgCBF7*) were identified from a cold-tolerant pomegranate cultivar. The overexpression of *PgCBF3* and *PgCBF7* led to enhanced cold tolerance in transgenic *Arabidopsis* plants under cold stress, as revealed by the increased concentration of proline and soluble sugar, as well as the decreased electrolyte leakage, MDA content, and ROS accumulation, the latter being associated with higher ROS scavenging by enzymes activity (CAT, SOD, and POD). Importantly, the signaling response to cold stress was elucidated in pomegranate, namely, PgICE1 activated the promoter activity of *PgCBF3* and *PgCBF7* via MYC recognition site and sped up the *PgCBF3* and *PgCBF7* transcripts. Moreover, the two pomegranate CBFs may mediate the expression of the downstream *Arabidopsis COR* genes in transgenic plants in response to cold stress. Taken together, *PgCBF3* and *PgCBF7* conferred higher cold tolerance in *Arabidopsis*, which provided new insights for enhancing cold tolerance using genetic engineering in perennial fruit trees.

## Data Availability

Not applicable.

## Supplementary Materials

The supporting information can be downloaded at: https://www.mdpi.com/article/10.3390/ijms23169439/s1.
