# Supplementary material for: Overexpression of PgCBF3 and PgCBF7 Transcription Factors from Pomegranate Enhances Freezing Tolerance in Arabidopsis under the Promoter Activity Positively Regulated by PgICE1
Source: Int J Mol Sci. 2022 Aug 21;23(16):9439. doi: 10.3390/ijms23169439 (PMC9408969; doi:10.3390/ijms23169439)
Supplement: Supplementary file 1 [file ijms-23-09439-s001.zip › Supplementary Material.pdf]

Figure S1 Full-length and deduced amino acid sequence analysis of pomegranate *PgCBF3* (**a**) and *PgCBF7* (**b**) genes

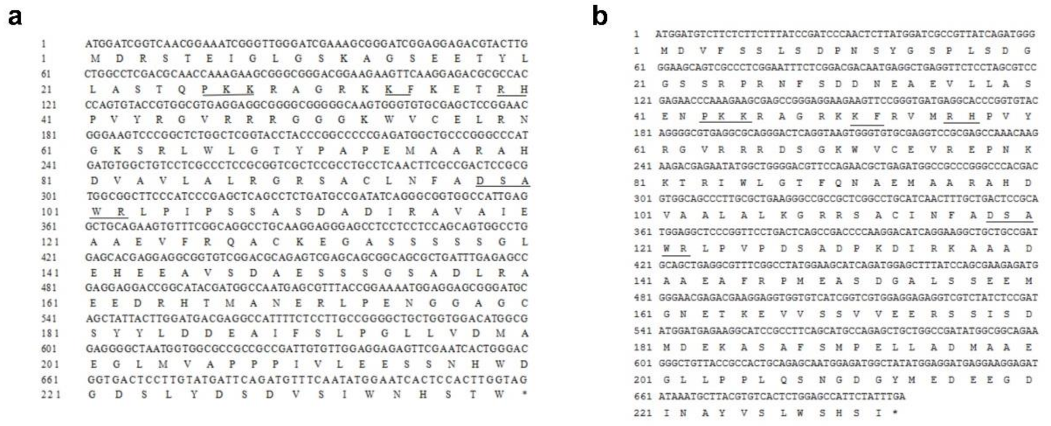

Figure S2 Alignment and phylogenetic analysis of PgICE1 protein with other ICE proteins and their function analysis

a

b

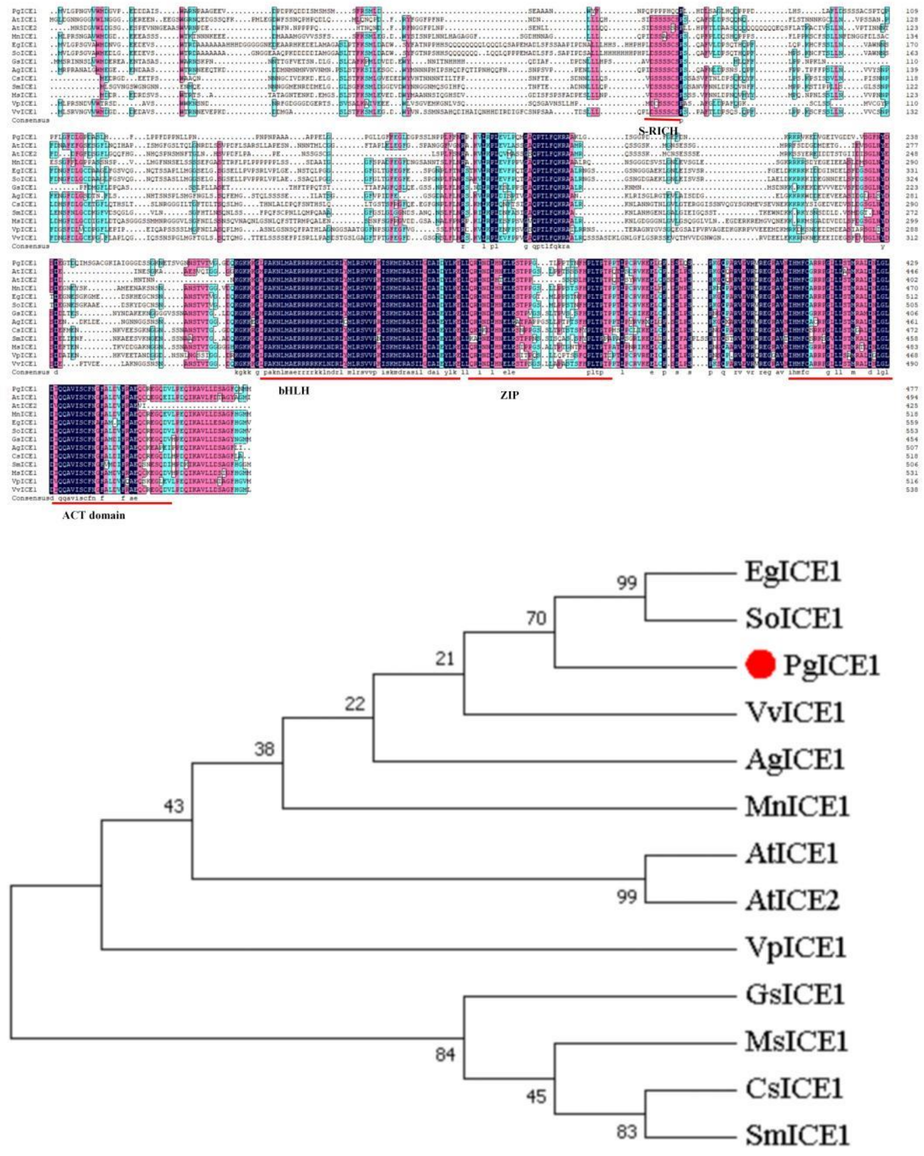

Figure S3 Promoter sequence analysis of the *PgCBF3* (**a**) and *PgCBF7* (**b**) genes

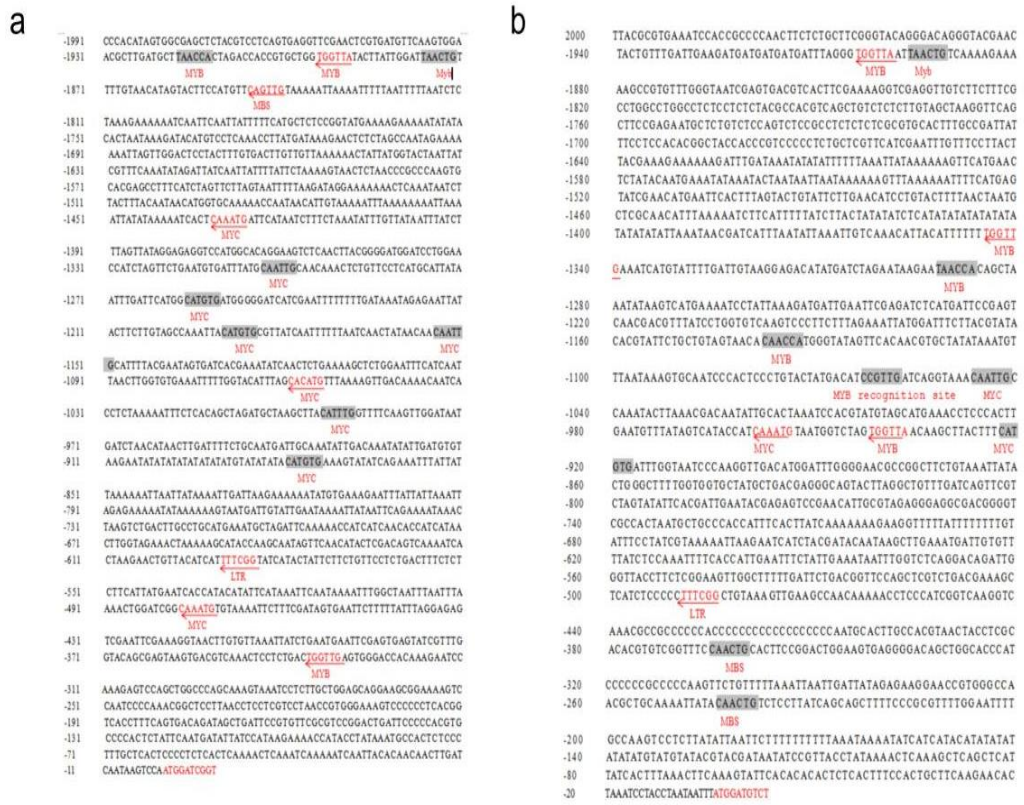

Table S1 Primers used for qRT-PCR

Gene name	Upstream primer (5'-3')	Downstream primer (5'-3')	GenBank Accession No.
PgActin	AGTCCTCTTCCAGCCATCTC	CACTGAGCACAATGTTTCCA	LOC116200207
*PgCBF*1	CCGCTATTACTTGGATGAGGA	AATCATACAAGGAGTCACCGT	PgL0134940.1
PgCBF2	GCACGCCTTACCGATACCA	TCCTTGAACACCACTCGCC	PgL0134950.1
PgCBF3	CAGCTATTACTTGGATGACGA	AATCATACAAGGAGTCACCGT	PgL0134960.1
PgCBF4	GCACCTACCCCACCGCTAAA	TGAGGCAGGCAGAGAGGCT	PgL0134980.1
PgCBF5	CCCGAAACCGTACGAGTCAG	GATGCTCCTCATCCGAGTGG	PgL0134970.1
PgCBF6	TTTCGAGTTGTCCCGAGGTG	ATGCCCCGAACAAGAAGGAG	PgL0201150.1
PgCBF7	AGAGATGGGGAACGAGACGA	AATGGCTCCAGAGTGACACG	PgL0233080.1
AtActin	GGTAACATTGTGCTCAGTGGTGG	AACGACCTTAATCTTCATGCTGC	AT3G18780
AtCOR15A	GGCGTATGTGGAGGAGAAAG	CCCTACTTTGTGGCATCCTTAG	AT2G42540
AtCOR47	GGCTGAGGAGTACAAGAACAA	ACAATCCACGATCCGTAACC	AT1G20440
AtKIN1	GCAATGTTCTGCTGGACAAG	TCCTTCACGAAGTTAACACCTC	AT5G15970
AtRD29A	GCTTTCTGGAACAGAGGATGTA	CGACTCTTCCTCCAACGTTATC	AT5G52310
PgICE1	CAGCGGGAAGAACGAGACTT	TAAAGCCGGTCGTTGAGCTT	LOC116214741

Table S2 Primer sequences for promoter, gene amplification, and vector construction

Primer name	Upstream primer (5'-3')	Downstream primer (5'-3')
PgCBF3	ATGGATCGGTCAACGGA	CCAAGTGGAGTGATTCCA
PgCBF7	ATGGATGTCTTCTCTTCTTT	AATAGAATGGCTCCAGAGTG
M13	CATTTTGCTGCCGGTCA	GTCCTTTGTCGATACTG
PgCBF3-2300	ACGGGGGACGAGCTCGGTACCATGG ATCGGTCAACGGA	GGTGTCGACTCTAGAGGATCCCCAAG TGGAGTGATTCCA
PgCBF7-2300	ACGGGGGACGAGCTCGGTACCATGG ATGTCTTCTCTTCTTT	GGTGTCGACTCTAGAGGATCCAATAG AATGGCTCCAGAGTG
pCAMBIA2300	GTAAGGGATGACGCACAATCCCAC	GCCGGTGGTGCAGATGAACTTC
PgCBF3-BD	AAGCTGATCTCAGAGGAGGACCTGC ATATGATGGATCGGTCA	TTATGCTAGTTATGCGGCCGCTGCAG CTACCAAGTGGAGTGATTC
PgCBF7-BD	AAGCTGATCTCAGAGGAGGACCTGC ATATGATGGATGTCTTCT	TTATGCTAGTTATGCGGCCGCTGCAG TCAAATAGAATGGCTCCAG
pGBKT7	GCGACATCATCATCGGAAGAG	CCTGAGAAAGCAACCTGACCTAC
PgCBF3 promoter	CCCACATAGTGGCGAGCT	CCCGATTTCCGTTGACC
PgCBF7 promoter	ATAGAAGAAGCAAGTGGCTCA	AGTTGGGATCGGATAAAGAA
PgICE1	CCTCCATTTCTTGTTCACCTCTC	AGAGAGAGAGAGAGAGCGAGCAG
pPgCBF3-0800	GGGCCCCCCCTCGAGGTCGACCCCAC ATAGTGGCGAGCT	TGTTTTTGGCGTCTTCCATGGTGGACT TATTGATCAAGTTGTTG
pPgCBF7-0800	GGGCCCCCCCTCGAGGTCGACATAG AAGAAGCAAGTGGCTCA	TGTTTTTGGCGTCTTCCATGGAAATTA TTAGGTAGGATTTAGTGTT
pB42AD	GTGCCAGATTATGCCTCTCCC	GAGACTTGACCAAACCTCTGGC
PgICE1-SAK	ATCCAAAGAATTCCCCGGTACCATGG TGCTGGGTCCCAAC	CATGATCTTTGTAATCCTCGAGTCAC ATCATATTTTGAAAGCCG
pPgCBF3-placzi	CTTGAATTCGAGCTCGGTACCCCCAC ATAGTGGCGAGCT	TGCCTCGAGGTCGACTGGACTTATTG ATCAAGTTGTTG
pPgCBF7-placzi	CTTGAATTCGAGCTCGGTACCATAGA AGAAGCAAGTGGCTCA	TGCCTCGAGGTCGACAAATTATTAGG TAGGATTTAGTGTT
PgICE1-pB42AD	GATTATGCCTCTCCCGAATTCATGGT GCTGGGTCCCAAC	AGAAGTCCAAAGCTTCTCGAGTCACA TCATATTTTGAAAGCCG
pSAK277	CATCGAAAGGACAGTAGAAAAGG	CATTAGAATGAACCGAAACCG
pGREENII0800	GTTTTCCCAGTCACGACGTT	GCCTTATGCAGTTGCTCTCC
